# Scale effects on the prediction of rare events in mature second-growth oak forests: a simulation study of cavity trees

**DOI:** 10.48130/FR-2021-0015

**Published:** 2021-08-26

**Authors:** Zhaofei Fan

**Affiliations:** School of Forestry and Wildlife Sciences, Auburn University, Auburn, AL 36849, USA

**Keywords:** classification and regression tree, logistic regression, resampling, computer simulation, the law of large numbers.

## Abstract

The abundance of cavity trees varies greatly due to the stochastic nature of cavity formation processes and involved disturbance agents. At small spatial scales such as a stand or plot, cavity tree abundance is extraordinarily difficult to predict precisely using tree and stand factors. In this study we used resampling methods to simulate the effect of spatial scale on cavity tree density (CTD) estimation using cavity tree data collected from a long-term forest experimental project. More than 53,000 measured trees were randomly divided into two approximately equal parts: the construction and test datasets, to construct classification and regression tree (CART) and logistic regression (LR) models to predict cavity probability and to test the accuracy of CTD estimation across varying spatial scales, respectively.

Simulation results showed that when the spatial scale was < 10 ha, the predicted CTD varied dramatically, and with this specific dataset, CART tended to overestimate, whereas LR and the sample mean method underestimated the true CTD estimated by the construction dataset. Compared with the sample mean method, the use of tree characteristics in both CART and LR resulted in slight or moderate reduction of the relative error (RE) (< 20%) when the spatial scale was < 10 ha. However, CART and LR, particularly CART, could improve CTD prediction efficiency significantly at larger spatial scales. For instance, the RE of CART was only 17% of the sample mean method at a spatial scale of 50 ha. Resource managers could use this information for cavity tree sampling and monitoring.

## INTRODUCTION

Cavity trees (either live or dead) are trees with holes or other structures large enough to shelter wildlife^[1]^. Formation of a cavity typically starts when an individual tree is killed or injured by disturbance events such as fires, insect attacks, diseases, animal excavations, and mechanical or chemical injuries^[2]^. Therefore, cavity tree probability is often weakly (but statistically significantly) associated with tree and stand characteristics, resulting in the dramatic variation of cavity tree abundance among different plots or stands, even those similar or alike in many aspects^[2−4]^. In the past, cavity tree prediction has been overwhelmingly confined to the level of a sampling or management unit such as a study plot or stand^[2, 5−7]^. The dramatic variation of cavity tree abundance at the plot or stand level and failure to explicitly consider the effects of spatial scale and sample size in cavity tree prediction models greatly limit the generality of the research findings^[4, 8]^.

Compared to the timber component, cavity trees as 'wildlife-related' components are relatively rare and vary dramatically by forest structure and over spatial scales/extents in forest ecosystems everywhere^[5−6, 9−12]^. Aforementioned random or semi-random disturbance agents associated with tree mortality and injury often exert significant impact on the distribution and dynamics of these components. Consequently, they are extremely difficult to predict by means of commonly used tree, stand, and site factors over the spatial scale or sample size for monitoring and predicting timber components or forest characteristics (e.g., forest fragmentation)^[13−16]^. Essentially, the prediction accuracy of rare components is greatly affected by three interrelated factors: relative frequency of subjects, sample size (spatial extent/scale), and the strength of associations between subjects and a set of predictor variables (represented by statistical models or rules)^[8, 17]^. Because information directly related to the frequency and location of rare components is typically difficult and costly to obtain, rare component occurrence/abundance is often modeled using predictor variables such as tree species, diameter at breast height, and decay class that are easily measured or less costly^[2, 4, 11−12]^.

The formation of a cavity in a tree or the likelihood for a tree to have cavities is essentially impossible to predict accurately in terms of its frequency or density at the sampling and management scale (e.g., a study plot or stand). Based on the law of large numbers, however, we can demonstrate that cavity tree density (CTD) is predictable at large spatial extents/scales that exceed a critical threshold specific to the target population to be sampled. As shown by Fan et al. ^[17]^, mean CTD across a landscape (e.g., > 4,000 ha) can be predicted with reasonable precision using a deterministic regression model based solely on stand age or stand size-class information, regardless of the fact that regression models consistently prove to be poor predictors of CTD at the plot or stand level^[2]^. To predict binary events such as whether a tree is a cavity tree or not, logistic regression (LR) and classification and regression tree (CART) methods have been widely used in forestry and ecological studies and reviewed in numerous literatures^[18]^.

Oak (*Quercus L.*) forests are among the most extensive and important forest ecosystems in North America^[19]^. In the eastern United States, oaks have been the most dominant forest species since the early Holocene^[20]^. In addition to timber production, oak forests provide both food and cover for a variety of wildlife species^[21]^. Wildlife-related components of oak forests such as cavity trees, snags (standing dead trees), down woody materials, and acorns have received widespread attention from wildlife biologists, foresters, and resource managers^[9, 21−23]^. The major objective of the study is to simulate the spatial scale effect on the prediction accuracy of CTD by using cavity tree data collected from a long-term, landscape-level study. Secondly, we will test how additional information (tree characteristics) and applications of cavity tree probability models (LR and CART) can improve the prediction accuracy of CTD across varying spatial scales/extents. This information will be helpful to cavity tree resource management and monitoring for wildlife and habitat conservation in such an important forest ecosystem under diverse management alternatives.

## METHODS

### Study site and the cavity tree data

**Figure 1:**
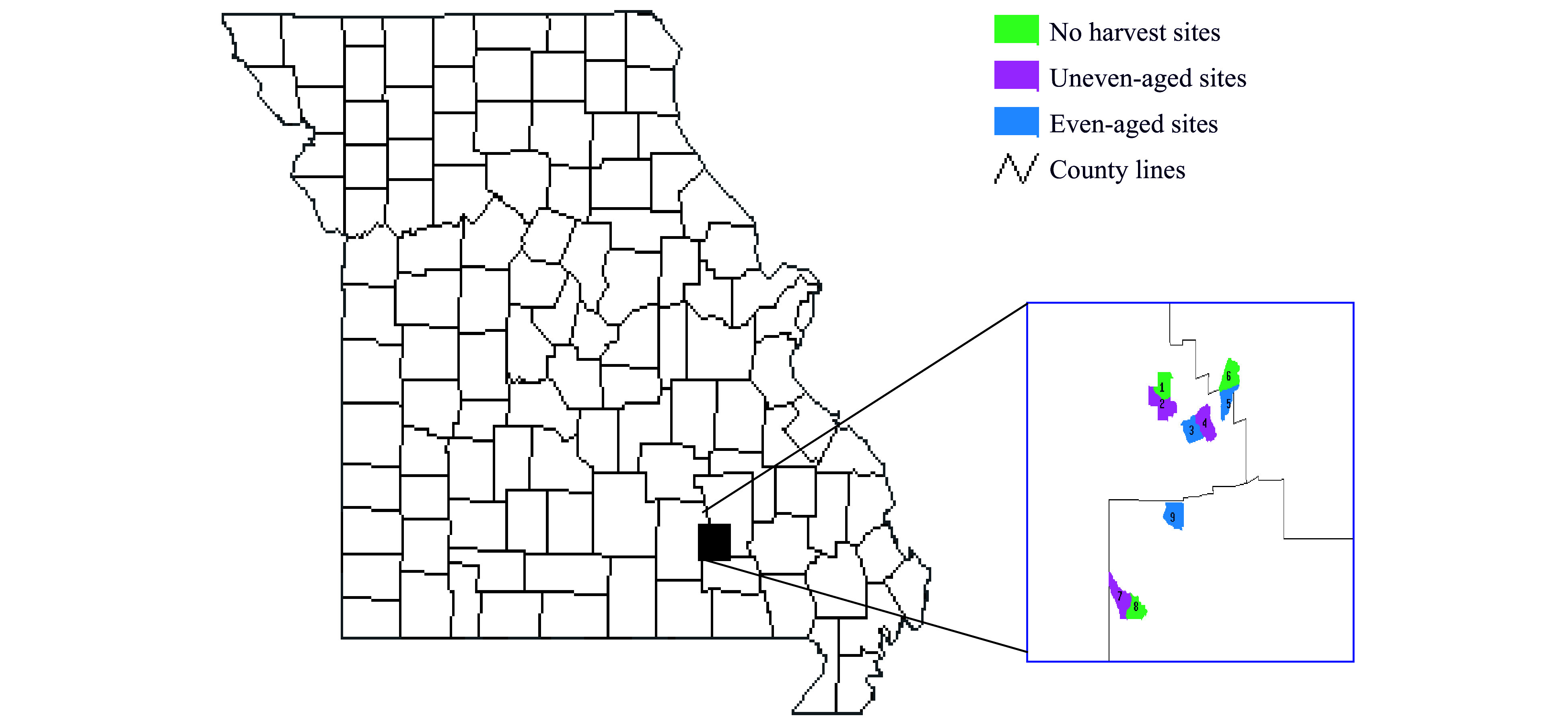
Location of the nine Missouri Ozark Forest Ecosystem Project (MOFEP) experimental sites. Sites range from 314 to 515 ha and are located in Carter, Shannon, and Reynolds County, Missouri, USA.

The Missouri Ozark uplands are dominated by second-growth oak-hickory and oak-pine forests which originated when native forests were heavily harvested in the early 1900s^[24]^. Since then, forests have experienced decades of partial harvesting and frequent low-intensity fires. White oak (*Quercus alba* L.), black oak (*Quercus velutina* Lam.), scarlet oak (*Quercus coccinea* Muenchh.), post oak (*Quercus stellata* Wangenh.), shortleaf pine (*Pinus echnina* Mill.), blackgum (*Nyssa sylvatica* Marsh.), and hickory (*Carya*) species account for over 94% of the forest canopy in terms of importance value. Stand ages mostly range from 70 to 100 years^[25]^. The woody vegetation inventory of the Missouri Ozark Forest Ecosystem Project (MOFEP) surveyed more than 50,000 individual trees > 11 cm dbh and associated environmental factors including slope, aspect, geo-landform, soil, and ecological land type (ELT) on 648 permanent 0.2-ha circular plots across the nine experimental sites which range from 314 to 516 ha in size both prior to and after treatment alternatives^[24, 26]^ (Fig. 1). The species, diameter at breast-height (dbh), crown class, decay class (for snags), and cavity presence/absence were recorded for each tree. A cavity for this study was defined as a hole with a diameter no less than 2.5 cm that appeared dark inside^[1]^. During the 1994−1995 inventory, prior to any harvest treatments, nearly 2,000 cavity trees were detected via ground-based observation. Later, a subset of trees was felled for further examination of cavity size and frequency^[1]^.

### Construction of CART and LR models to predict cavity tree probability

In a previous study^[11]^, we used CART and LR^[27]^ to analyze the statewide FIA data to uncover tree factors and predict the probability of cavity trees in Missouri second-growth forests as well as in old-growth stands of four Midwest states (Missouri, Illinois, Indiana, and Iowa). In the hierarchical CART profile, individual trees were grouped into different nodes based on tree characteristics and thus, the whole sample was split into a set of strata (nodes) that differed in cavity tree probability (or frequency). Effects of species (group), decay class, diameter at breast height (dbh) and interactions on cavity tree probability were evaluated explicitly by CART. As stated in our previous analysis, however, the lack or scarcity of observations (cavity trees) in the combinations of certain species group and decay class (particularly decay classes VI and VII) precluded the simultaneous analysis of main factors and their interaction effects through logistic regression. Therefore, logistic regression models that include only the main terms (without interactions) will be constructed for tree species (groups) and decay classes on the basis of the CART model^[4]^.

**Figure 2:**
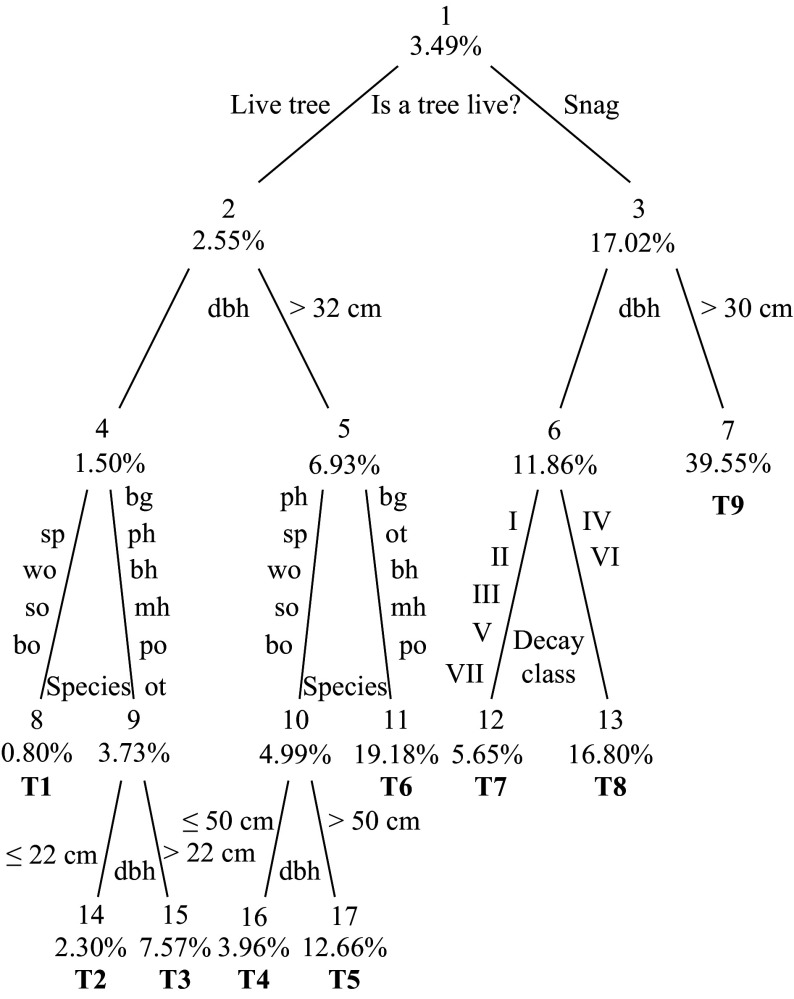
The classification and regression tree (CART) classifier of cavity tree distribution based on tree attributes. Nodes are numbered 1 through 17, and terminal nodes have the additional labels T1 through T9. Groups of nodes (nodes 2, 3; nodes 4, 5, 6, 7; nodes 8, 9, 10, 11, 12, 13; and nodes T1 through T9) represent strata in a hierarchical system of the CART model based on tree status (live or dead), diameter at breast height (dbh), species, and decay class.

**Figure 3:**
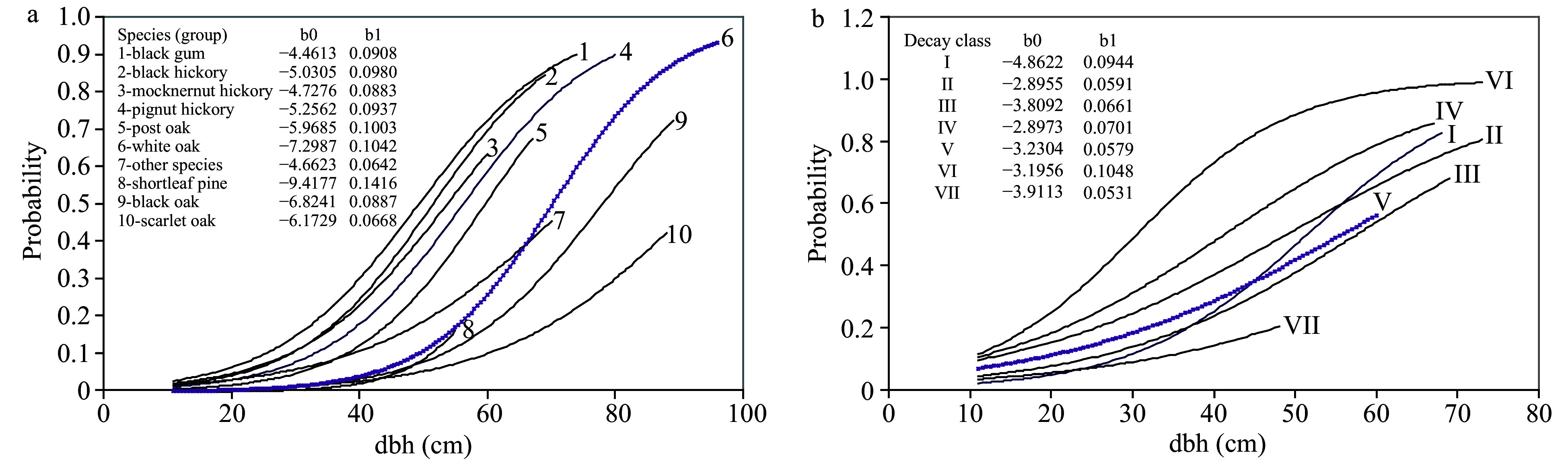
Change in the probability that a tree bears at least one cavity predicted by the logistic regression (LR) model for (a) live trees and (b) dead trees. The estimated regression coefficients for the LR models were listed besides the fitted probability curves.

To construct a CART model to predict or estimate the CTD based on individual tree characteristics, we randomly divided the cavity tree data (including 53,338 trees > 11 cm dbh, 1,899 of them being recognized as cavity trees) into two parts: construction and test sets. We used the construction set (25,725 trees, 897 of them as cavity trees) and the 10-fold cross-validation method to construct the single 'best' CART model. The best CART model classified sample trees into nine (terminal nodes 7, 8, 11, 12, 13, 14, 15, 16, and 17) strata hierarchically based on decay class, dbh and species groups with cavity tree probability ranging from < 1 to 40% (Fig. 2). We further constructed the LR model to predict cavity tree probability using dbh as predictor for ten species or species group of live trees and seven decay classes of snags, respectively (Fig. 3). Fan et al. (2003)^[4]^ described the construction details of CART and LR models in the context of cavity tree probability prediction.

### Prediction of CTD using the CART, LR, and sample mean (design-based) method

Given the estimated cavity tree probability within a stratum in CART, one solely needs to estimate/sample tree numbers for each specified stratum to predict/monitor the CTD. The CTD within a specific area of A-ha which occupied by n trees can conveniently be estimated, via CART, as the mean of a stratified sample

1 $ { C}\hat { T}{ D} = \sum\limits_{i = 1}^k {{n_i}{p_i}_{}} /A $

where n_i_ and p_i,_ are the tree number and cavity tree probability of terminal node (stratum) i of the CART model with k (here, k = 9) terminal nodes in total, and satisfy $\sum\limits_{i = 1}^k {{n_i} = n} $ and $ 0 \leqslant {p_i} \leqslant 1 $, respectively. Likewise, the CTD within a specific area of A-ha occupied by n trees can be estimated deterministically, via LR model, as,

2 ${ C}\hat { T}{ D} = \sum\limits_{i = 1}^n {{p_i}} /A $

where *p*_*i*_ is the predicted probability tree *i* being a cavity tree through the logistic regression model. With the sample mean (design-based) method, the CTD can be estimated as,

3 $ { C}\hat { T}{ D} = 0.0349\times n/A $

When n or A is small, the estimated p_i_ and subsequently, the estimated CTD, vary dramatically due to the stochastic nature of the related abiotic and biotic factors that affect cavity formation (e.g., disease, animal excavation, mechanical damage, species composition, dbh, and decay class). However, based on the law of large numbers, as n or A increases, both the estimated p_i_ and CTD will converge to a level corresponding to the forest-wide average for the forest condition and disturbance regime. Therefore, to effectively predict the CTD or cavity tree abundance for a specific forest type or ecoregion, n or A should be large enough that the estimated p_i_ or CTD (i.e., $ {\hat p_i} $ or ${ C}\hat { T}{ D} $) differs from the true value by a relatively small amount, ε. Specifically,

4 $\mathop {\lim }\limits_{n \to \infty } P[|{\hat p_i} - {p_i}| < \varepsilon ] = 1$

or

5 $\mathop {\lim }\limits_{A \to \infty } P[|{\rm C}\hat {\rm T}{\rm D} - {\mathrm{CTD}}| < \varepsilon ] = 1$

With the CART- or LR-predicted CTD, it is extremely difficult to use a closed form to quantify the relationship between ε and n or A because of the changes of species composition and forest structure over space. We used computer simulations to describe empirically how ε, a random variable driven by tree and stand structure, varies with n or A.

The true CTD for a specific area is often unknown. In the simulation we used the calculated CTD from the test dataset (CTD_calculated_) to replace the true CTD and the relative error (RE) defined in equation (6) to replace ε to quantify the prediction accuracy of the CART and LR model and the sample mean (design-based) method. RE measures how close the CART- or LR-predicted CTD (CTD_model-predicted_) is to CTD_calculated_ over a specific spatial scale (A) or sample size (n) and takes the form

6 $ RE = \left| {1 - \frac{{{{CT}}{{ D}_{ {\mathrm{model - predicted}}}}}}{{{{CT}}{{ D}_{\mathrm{calculated}}}}}} \right| $

### Simulating scale effects on the RE of CTD prediction via CART and LR models

As stated above, due to the great heterogeneity (species, size, stocking, etc) of mature oak forests over space, it is more informative to use the virtual spatial area A_v_ instead of the real spatial area A (usually unknown) to gauge the change in the RE over the spatial scale (area). We first calculated the stocking percent of each sampled tree from the test data set (27,613 trees > 11 cm dbh, 1,002 of them as cavity trees) based on tree species and dbh (cm)^[28−30]^,

for oaks and hickories,

7 ${{ StockPct }}= 0.1\times\left\{ - 0.0507  +  \left(\frac{{0.1698\times dbh}}{{2.54}}\right)  +  \left[0.0317\times{\left(\frac{{dbh}}{{2.54}}\right)^2}\right]\right\} $

for short leaf pine,

8 $ {{ StockPct }} = 0.1\times\left\{ 0.08798 + \left(\frac{{0.09435\times dbh}}{{2.54}}\right) + \left[0.0253\times{\left(\frac{{dbh}}{{2.54}}\right)^2}\right]\right\} $

for all other species,

9 $ {{ StockPct }} = 0.1 \times\left\{  - 0.17979  +  \left(\frac{{0.21425 \times  dbh}}{{2.54}}\right)  +  \left[0.01711 \times {\left(\frac{{dbh}}{{2.54}}\right)^2}\right]\right\} $

We assumed the independence of the trees (the probability of a tree to be a cavity tree does not change with other trees) and drew a simple random sample (SRS) of size n (n = 10, 20, 30, …) from the test tree data set to make up a set of virtual plots of different sizes (A_v_). The size (A_v_) of a virtual plot was calculated as the sum of the stocking percentage multiplied by the expansion factor of all trees within it. The RE was calculated for different sizes of A_v_ based on equation (6) for the CART model, LR model, and the sample mean (designed-based) method with a mean cavity tree probability of 0.0349 (the root node of the CART model in Fig. 2). We ran the above process 100 times by choosing different random numbers and calculated the mean RE and the standard error corrected based on the finite population correction coefficient corresponding to different scales of A_v_. To evaluate the efficiency of the CART and LR models relative to the sample mean (design-based) estimation, which did not use any tree information (e.g., tree status, dbh, species, decay class) at a specific spatial scale, we calculated the relative efficiency of a model as,

10 ${{ REF F}} = \frac{{R{E_{\mathrm{model - based}}}}}{{R{E_{\mathrm{design - based}}}}} $

## RESULTS AND DISCUSSIONS

The CART model in Fig. 2 identifies four variables that are associated with cavity tree probability: (1) whether the tree is live or dead (nodes 2, 3), (2) DBH (nodes 4−7, 14−17), (3) tree species (nodes 8−11), and (4) snag decay class (nodes 12, 13). The root node (node 1) represents the whole tree sample with mean cavity tree probability of 0.0349, a design (sampling)-based estimate of cavity tree abundance. The CART classifier illustrates the relative importance of each variable for estimating cavity tree probability. For example, tree status (whether sampled trees are alive or dead) is most important; cavity tree probability (17.02%) within the dead trees is nearly 7 times greater than that (2.55%) within the live trees. The left side of CART reveals the probabilistic distribution of cavity trees by dbh and tree species (group) among live trees, while the right side illustrates cavity tree probability by DBH and decay class among snags (standing dead trees). In relation to the nonparametric CART model, the logistic regression model quantifies how cavity tree probability changes with dbh for different tree species of live trees (Fig. 3a) and decay classes of dead trees (Fig. 3b) in a parametric way, with a group of regression coefficients estimated explicitly for dbh by tree species and decay class, respectively.

**Figure 4:**
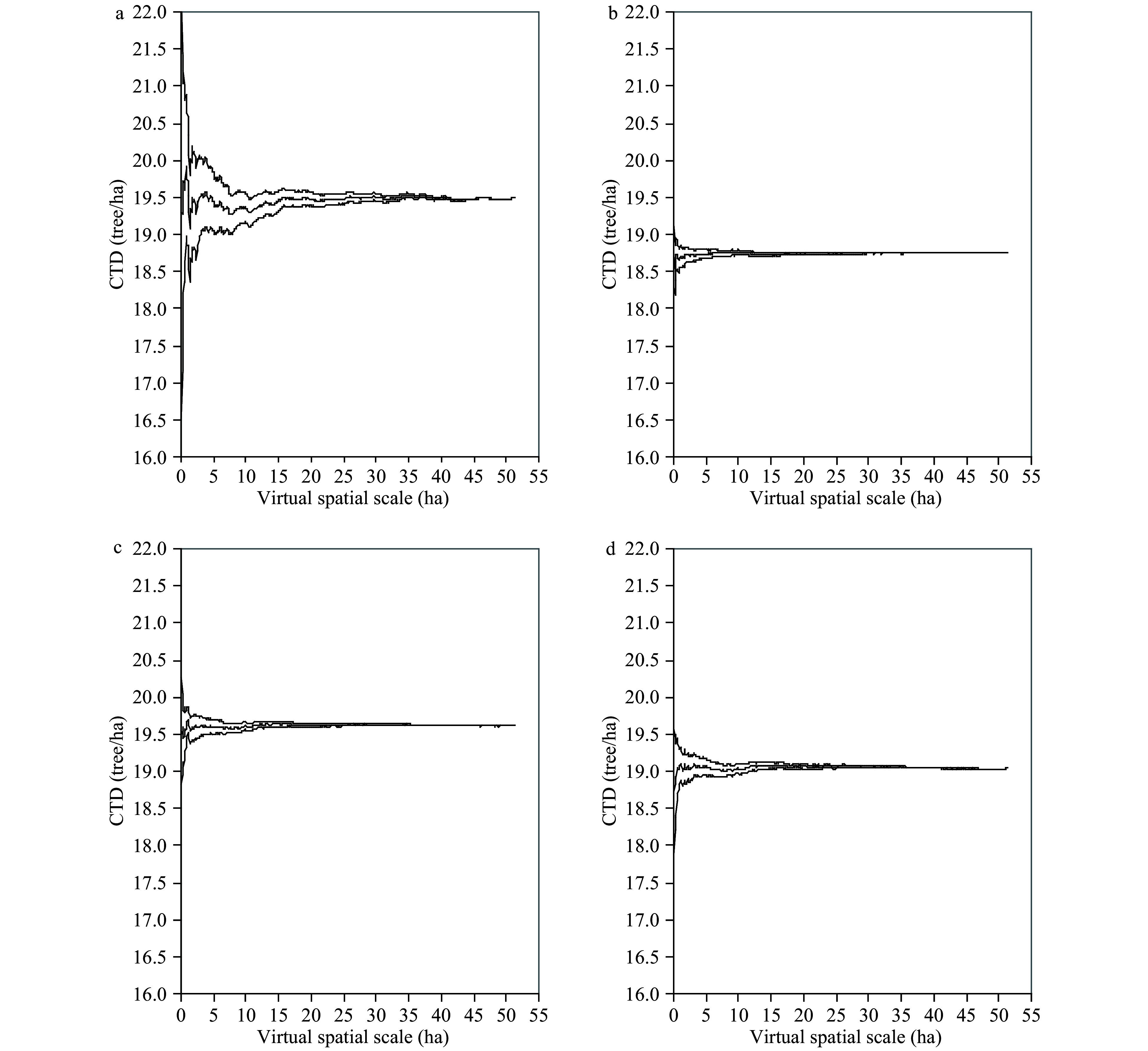
Changes in the mean CTD and 95% CI with virtual spatial scales: (a) test dataset; (b) sample mean (design-based) method; (c) CART model; (d) LR model. The mean CTD and 95% CI were estimated based on 100 simulation runs.

Fig. 4 shows the changes in the CTD as well as 95% confidence intervals (CI) across different virtual spatial scales, A_v_. The true CTD in the test dataset varied dramatically and had a wider CI when A_v_ was less than 5 ha and then gradually stabilized (Fig. 4a). Changes of the estimated CTD based on the sample mean method (using the construction dataset), CART, and LR followed similar patterns, but the estimated CTD had a relatively narrower CI (Fig. 4b, c, and d) due to the 'smooth effect' of different models. Across spatial scales, the CART-predicted CTD tended to overestimate the true CTD, but the LR and sample mean method underestimated the true CTD. For small samples, it was possible to greatly over- or under-estimate the true CTD, but at large scale the bias will be stabilized.

Compared to LR, the CART classifier developed here can be easily applied with any standard forest inventory system, including the statewide forest inventory and analysis (FIA) sample of forest resources^[13]^. Although our data are limited to second-growth mature upland oak forests in the Missouri Ozarks, the same suite of independent variables have been found to be relevant for cavity estimation for state-wide cavity tree estimates based on FIA samples^[11]^. Nevertheless, applications of the CART model (Fig. 2) to other forest types such as coniferous forests should be done prudently considering the fact that the model was developed with inventory data from sites that were mature and relatively undisturbed^[1, 25, 31−32]^. Forests with different species compositions, age/size structures, or disturbance histories may have different cavity probabilities and/or classification thresholds^[12, 33]^. Cavity probabilities or classification thresholds can also be affected by differing definitions of what constitutes a cavity tree. The MOFEP data tallied cavity trees having at least one cavity at least 2.5 cm in diameter. A cavity size-threshold larger than 2.5 cm would generally decrease the probability of cavity tree occurrence for a given class of trees.

**Figure 5:**
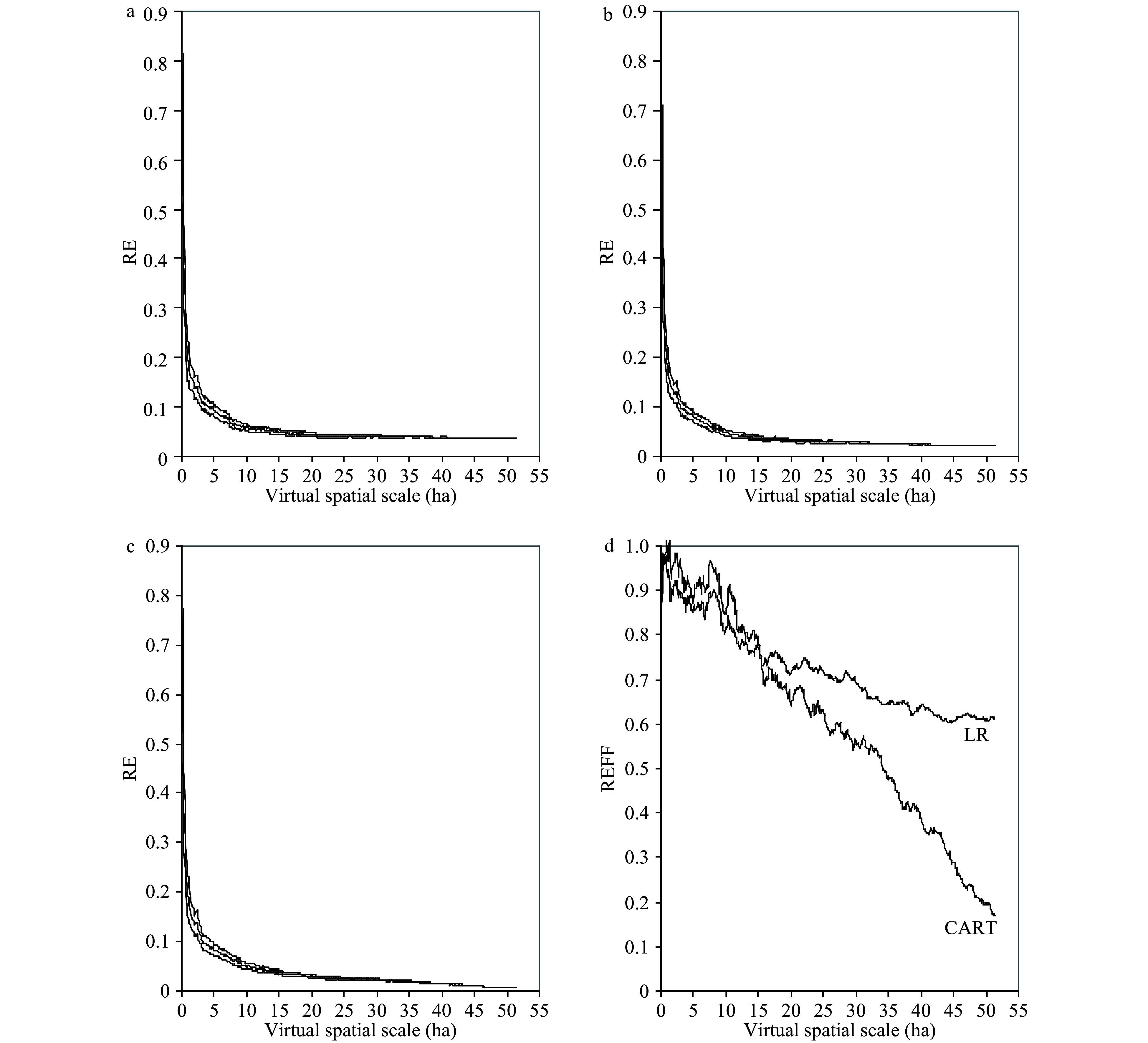
Changes in the mean relative error (RE) and 95% CI with virtual spatial scales: (a) sample mean method; (b) CART model; (c) LR model; (d) relative efficiency (REFF) of the CART and LR models relative to the sample mean (design-based) method. The change of REFF with spatial scales in panel (d) showed that compared with the LR model and the sample mean (design-based) method, the application of CART model and additional tree information could greatly reduce relative errors in CTD prediction at large spatial scales (sample sizes).

The RE and 95% CI of the estimated CTD decreased significantly with virtual spatial scale until 5 ha (Fig. 5). When the virtual spatial scale was larger than 5 ha, the RE values for the sample mean method (Fig. 5a), CART (Fig. 5b), and LR model (Fig. 5c) were all less than 10%. Across all spatial scales, CART and LR more accurately estimated the CTD than the sample mean method, as further information on tree species, dbh, and decay class was employed. The REFF for both CART and LR continually decreased with the spatial scale (Fig. 5d). However, CART was more accurate than LR in this study largely due to the 'instability' or 'sensitivity' nature of CART^[27]^. For example, at a spatial scale of 50 ha, the RE of CART and LR were, respectively, about 17% and 65% of that for the sample mean methods. But at small scales, the improvement of the prediction accuracy of both CART and LR was limited because of the significant stochasticity of the cavity presence-tree characteristic relationship as shown by model parameters (Fig. 2 and Fig. 3).

These results illustrate two important points from the perspective of monitoring cavity trees or predicting the number of cavity trees per ha. First, estimates for areas smaller than about 5 ha will have low precision, and there is little gain in precision at small scales such as less than about 10 ha. Second, reliable cavity tree estimates can be derived from very simple information − namely the number of trees that fall into the nine strata (terminal nodes). This type of information can be rapidly collected, even when the total area sampled exceeds 10 ha. Various types of line or strip sampling are particularly well suited to rapid collection of this type of data. Moreover, sufficient information is included in nearly all timber or vegetation inventories^[13]^.

The impact of the spatial scale on sample estimates and model predictions of rare events such as cavity trees was largely overlooked but critical to understand ecosystem function and processes^[34−37]^. Sample surveys for cavity tree estimation and other rare components were often simply piggy-backed onto inventories designed to estimate other components of the forest canopy such as stand density and basal area. Consequently, reported descriptive statistics for the CTD might provide little information due to the large variation among plots and low precision of estimates^[8, 17]^. Likewise, regression and similar statistical models of cavity tree density applied to individual plots or stands were less informative due to their large residual errors at those spatial scales^[2]^.

Our findings have important implications for forest managers seeking information about the CTD. Typical forest inventory schemes that measure a small proportion (< 10 ha) of the total area of a forest tract (e.g., those designed for timber inventory) are unlikely to produce reliable CTD estimates. Moreover, collecting additional information such as whether the tree is alive, species group, dbh, or decay class will not greatly improve the CTD estimate for small tracts. For small (< 10 ha) forest tracts where precise estimates of CTD are needed, managers should consider a specialized sampling scheme or enumerating cavity trees. Similarly, forest tracts ranging from 10 to 400 ha may require a greater sampling intensity than is commonly used by forest managers. The intensity of a given forest inventory varies with its purpose, but timber volume inventories generally encompass less than 5% of the forest area. Our analysis indicates that, for forest tracts larger than 400 ha, traditional inventory methods used to estimate timber volume may well be adequate for estimating the CTD.

This study also demonstrates the importance of large-scale experimental forest research programs. Although studies such as MOFEP are costly and continue for many decades, they provide invaluable data that are difficult to obtain otherwise. Such data are essential for the systematic study of scale issues through computer-intensive methods (simulation) as carried out in this study. Computer simulation can rapidly offer insights into ecosystem dynamics and responses to management alternatives^[8]^ as well as help refine current research hypotheses or establish new ones. Ecosystem components, particularly rare and/or disturbance-associated components, are sensitive to scale. Computer-based multi-scale simulations may provide a clearer picture of the involved factors and interactions associated with scale transitions. Moreover, they help identify crucial scales for ecosystem modeling and sampling that would be difficult to discover by the experimental/survey research alone^[8]^.

In this study, a single model or classifier approach was used to predict the CTD. However, the ensemble approach that uses hundreds or thousands of classifiers/models such as the randomForest algorithm should be able to further improve the prediction accuracy. The ensemble of classifiers can fully explore the information embedded in the sample and be combined to 'vote' on a best estimate of new samples^[38−39]^. Simulating the scale effect on rare events through the ensemble of classifiers and models is beyond the scope of this study and will not be discussed here.

## CONCLUSIONS

For oak forests of the Missouri Ozarks, CART analyses can be used to estimate the probability that a given tree at least 11 cm in dbh will have at least one cavity based on the following independent variables, in order of importance: tree status (i.e., live or dead), dbh, species, and decay class (dead trees only). However, such estimates are imprecise when applied to trees on one or a few inventory plots because cavity trees are relatively rare, and their occurrence is highly variable. However, when CART models are applied to populations of trees to estimate mean cavity tree abundance, the precision will be suitable for many practical applications. Through a series of simulation analyses, we determined that, when the total sampled forest area exceeds 10 ha, the relative error of the mean cavity tree abundance was consistently less than 20%. The relative error of the estimated CTD will decrease dramatically with sampled areas within the range of 0 to 10 ha; however, additional gain in precision will increase slowly when the sampled area exceeds 10 ha.

Measuring all four independent variables results in the most precise estimate of the CTD, but the hierarchical nature of the CART model accommodates very simple inventory schemes. A simple tally of the number of live and dead trees is sufficient to utilize the CART model for estimating the mean number of cavity trees. However, for a fixed level of precision, the total sampled area for a simple live/dead inventory will need to be greater than that of a more complex inventory that also tallies tree dbh class, species group, and snag decay class. The model-based sampling scheme can be readily applied to estimate cavity tree abundance with FIA data and other forest inventories, provided the total sampled area is appropriate for the desired level of precision. This general approach to model-based sampling appears to be amenable to other classes of highly stochastic, rare events (or categories) such as tree mortality or tree damage from exogenous disturbances.
